# Food Fraud Implications and Regulatory Challenges in South Africa: A Review

**DOI:** 10.3390/foods15081282

**Published:** 2026-04-08

**Authors:** Likentso Sylvia Shuping, Kgomotso Lebelo

**Affiliations:** 1 Department of Environmental and Occupational Studies, Faculty of Applied Science, Cape Peninsula University of Technology, Cape Town 8000, South Africa; 2 Occupational and Environmental Exposure and Health Division, School of Public Health, Faculty of Health Sciences, University of the Witwatersrand, Johannesburg 2193, South Africa

**Keywords:** food fraud governance, food policy and regulation, informal markets, counterfeit products, public health risks, South Africa

## Abstract

Food fraud has emerged as a significant and under-recognised public health threat, with documented global incidents resulting in severe illness, hospitalisations, and fatalities. International estimates suggest that up to 9% of the global food trade is adulterated. In South Africa, evidence of mislabelling, substitution, counterfeit products, illicit trade, and the use of unauthorised additives continues to surface, yet the national burden and regulatory response remain insufficiently characterised. This review synthesised peer-reviewed literature and articles from reputable South African media sources published from 2015 to December 2025, focusing on food fraud within the South African context. Searches were conducted across Web of Science (WoS), Scopus, and PubMed, supplemented by Google Scholar and the EU Food Fraud Database, with emphasis on studies reporting fraud associated with South African food products. Standard PRISMA procedures guided the final selection of fifteen (14) eligible articles. These studies reveal widespread food fraud driven mainly by economic gain. Common practices include substituting high-value products, mislabelling meat and seafood, altering dates on expired goods, and producing counterfeits with unauthorised additives and packaging. Collectively, these factors compromise consumer health, undermine industry integrity, and impede effective surveillance. Strengthening South Africa’s food fraud prevention ecosystem will require coordinated multisectoral engagement, targeted investment in detection technologies, and robust regulatory reforms.

## 1. Introduction

**Figure 1:**
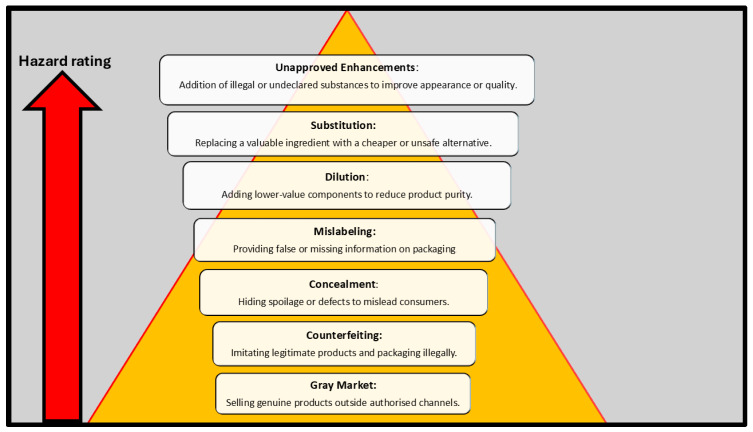
Hazard-based ranking of food fraud concepts based on severity and potential public health impact. The model categorises seven common food fraud types, i.e., unapproved enhancements, substitution, dilution, concealment, mislabelling, counterfeiting, and grey market, according to their relative risk. Higher tiers represent a greater potential for consumer harm due to toxicity, allergen exposure, or deception, while lower tiers reflect economic or regulatory concerns with minimal direct health risk.

Food fraud, while historically recognised, has re-emerged as a critical challenge within modern food systems due to increasingly complex supply chains, economic pressures, and evolving fraudulent techniques. Globally, the practice encompasses a spectrum of intentional deceptive activities, such as adulteration, mislabelling, substitution, counterfeiting, grey-market trading, concealment, and the use of unauthorised additives, carried out for economic gain at the expense of consumer safety [1,2,3]. Fundamentals of food fraud are illustrated in Figure 1, which is a conceptual risk hierarchy linking fraud types to public health severity. The illustration depicts various forms of food fraud. The occurrence differs between developed and developing countries, owing to greater detection of food fraud in the former and measures in place to mitigate it. Food fraud is used as an overarching construct, within which adulteration, substitution, mislabelling, and counterfeiting are classified as distinct but related phenomena. Spink and Moyer [2] define food fraud as “a deliberate and intentional substitution, addition, tampering or misrepresentation of food, food ingredients or product for economic gain”. Robson et al. [4], in a study reviewing food fraud terminology, found that most peer-reviewed studies defined food fraud as intentional deception for economic gain involving food, whereas mislabelling and food adulteration are types of food fraud. Economically motivated adulteration is also a subcategory of food fraud, often defined as the fraudulent, intentional substitution or addition of a substance in a food product to increase its value or reduce production cost [5]. Table 1 shows the extent of the problem in Sub-Saharan Africa. Estimates suggest that up to 9% of the global food trade may be fraudulent, reflecting the scale of the problem and the high financial incentives that drive it [6]. According to Hoffman et al. [6], the estimated annual cost of food fraud in the food manufacturing industry is approximately $30–$40 billion. While high-profile incidents such as the melamine adulteration in China, methanol poisonings in India and Indonesia, and the horse meat scandal in Europe have catalysed international attention, the actual global burden remains significantly underreported [4,7]. Globalisation, or international food trade, has enabled food fraud to flourish. This is due to the numerous times food changes hands as it passes from farmers, producers, manufacturers, processors, brokers, and distributors [8]. The challenge in detecting adulterated food products lies in consumers’ ability to determine if a product is adulterated, which often occurs after they have consumed it. Food adulteration as a form of food fraud may result in detrimental health effects, such as food poisoning, allergic reactions [9,10], and illnesses such as asthma, ulcers, kidney failure, gastrointestinal problems, and cancer, to name a few [11].

Some of the global incidents include a food adulteration incident that occurred in 1981 in Spain, where fuel oil for industrial use was sold as olive oil for human consumption, which resulted in 1200 deaths and 20,000 hospitalisations. In 2008 and 2009 in India, cases of methanol intoxication resulted in 319 total deaths [7]. In 2008, in China, melamine was added to milk to increase protein content, which resulted in the death of 6 babies [6,12]. In Indonesia, 25 people died after consuming fermented palm wine in 2009. An outbreak of methanol poisoning occurred in Rafsanjan (Iran), which resulted in 8 deaths and 694 people affected. Horse meat that was labelled as beef was detected in the UK and Ireland in 2013, and in Nigeria, 89 people died from toxic alcoholic beverages in 2015 [7]. Food fraud is an old problem that continues to escalate despite its true prevalence remaining uncertain.

High-income regions such as the European Union, the United States, and parts of Asia have responded by implementing robust surveillance infrastructures, including the EU Rapid Alert System for Food and Feed (RASFF), the US FDA’s Food Fraud Databases, blockchain-based traceability systems, and advanced analytical authentication technologies [11,13]. These platforms enable real-time reporting, coordinated regulatory responses, and the integration of sophisticated detection tools, including DNA-based species identification, spectroscopic fingerprinting, and untargeted metabolomics [14]. As a result, food fraud in these regions is systematically detected, documented, and publicly communicated.

By contrast, South Africa remains substantially under-researched, with the scientific literature limited to scattered studies that focus primarily on species substitution in meat and seafood, isolated cases of counterfeit packaged foods, and occasional reports of excessive chemical residues flagged through international export systems. Several structural and socioeconomic realities contribute to this gap. These include a large and dynamic informal food economy, variability in enforcement capacity across municipalities, inadequate laboratory resources for routine food authentication, and regulatory penalties that are disproportionately low relative to the severity of offences [15]. Despite growing public concern, particularly following incidents involving chemically contaminated snacks, mislabelled meat products, and counterfeit beverages, South Africa lacks an integrated national food fraud monitoring system. Consequently, many cases go undocumented or unresolved, and the full extent of food fraud in the country remains largely unknown.

From a conceptual standpoint, the South African context has not been adequately examined through established theoretical frameworks that dominate global food fraud research. Models such as the Food Fraud Vulnerability Assessment (FFVA), the Food Fraud Prevention Cycle (FFPC), and broader theories of Economically Motivated Adulteration (EMA) offer structured approaches to understanding fraud drivers, identifying vulnerabilities, and implementing prevention-focused controls [4,15]. However, very few South African studies explicitly apply or evaluate these frameworks, resulting in fragmented insights and limited alignment with international best practice. The public health implications of this evidence gap are profound. Food fraud exposes consumers to toxic chemicals, undeclared allergens, pathogenic contamination, and products of inferior nutritional quality [16,17]. These risks disproportionately affect low-income and rural communities that depend on informal markets where fraudulent products circulate with minimal oversight [16,17]. Beyond health concerns, food fraud undermines consumer trust, erodes the integrity of the national food industry, and poses reputational risks to South African exports, an area on which the country relies heavily on international trade.

Given these gaps, there is an urgent need for a consolidated and critical synthesis of food fraud research conducted in South Africa. This review synthesises peer-reviewed articles published between 2015 and 2025 to map fraud patterns, assess affected commodities, evaluate detection methods, and identify systemic regulatory vulnerabilities. By situating South Africa’s challenges within a broader global and theoretical framework, the review aims to clarify the current state of knowledge, highlight critical shortcomings, and inform future research, policy development, and enforcement strategies. Table 1 provides types of food fraud activities identified in Sub-Saharan Africa, including South Africa.

**Table 1:** Examples of food fraud incidents in Sub-Saharan Africa [16].

Country	Type of Food Fraud	Food Categories	Description
Burkina Faso	Illegal export	Cereals and nuts	600 tonnes of grains, maize, and nuts were seized
Burundi	Illegal trade	Dried food	Parchment of coffee
Cameroon	Counterfeit	Honey	Production of fake honey using sugar
Ghana	Adulteration	Oil	Addition of Sudan IV dye in Palm oils
Ivory Coast	Smuggling	Fruits	35 tonnes of contraband cocoa seized in Ghana
Kenya	Counterfeit	Alcoholic beverages	300 cartons of counterfeit spirits were confiscated
Mozambique	Illegal fishing, misrepresentation of origin, and falsified documents	Seafood	Four tonnes of fish were illegally caught
Namibia	Counterfeit	Alcoholic beverages	Dismantled 120 illegal distilleries and seized over 6000 litres of smuggled liquor
Nigeria	Smuggling	Cereal	Over 90,000 bags of 50 kg of rice were seized
Rwanda	Adulteration	Honey	Addition of sugar syrup and crushed yellow bananas to the honey
South Africa	Illicit trade	Seafood	Poaching and the illegal trade of abalone
Tanzania	Counterfeit	Alcoholic beverages	Production and sale of illicit alcoholic beverages
Uganda	Artificial enhancement	Poultry and meat	Chicken and pigs were fed with anti-retroviral drugs to accelerate growth
Zimbabwe	Counterfeit	Seeds	Fake maize seeds seized

## 2. Methodology

The literature search was conducted in online databases, including Web of Science (WoS), PubMed, Google Scholar, and Scopus, from 2015 to 2025 to ensure the inclusion of the most recent information. Data extraction and quality assessment were conducted by two authors, who independently performed the tasks. Both authors cleared discrepancies and duplicates for quality assurance. For each included study, data on the food product tested, type of fraud, analytical method, and key findings were independently extracted by two reviewers.

### 2.1. Eligibility Criteria

**Figure 2:**
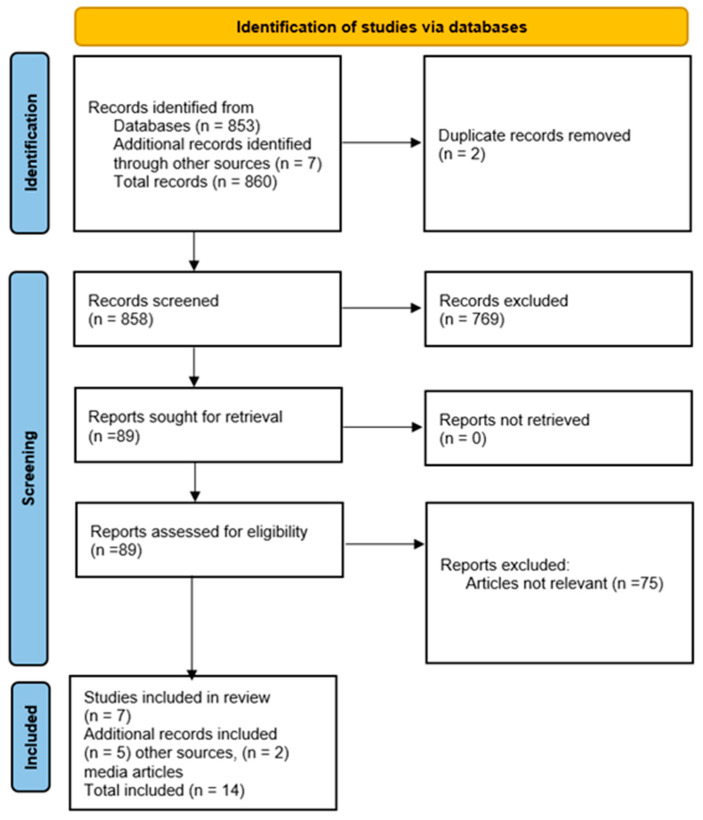
PRISMA 2020 flow diagram for systematic reviews.

The following inclusion and exclusion criteria were used: articles written in English, from peer-reviewed full-text reviews, and original research from 2015 to 2025. In addition, reports from reputable South African media platforms (news channels) were also included, as were those from the RASFF food fraud database, which reports food fraud within South Africa or involving South African food exports. Unpublished papers, non-English written articles, abstracts, dissertations, conference proceedings, books, and book chapters were excluded. The screening, evaluation, and identification of records for eligibility were conducted in accordance with the Preferred Reporting Items for Systematic Reviews and Meta-Analyses (PRISMA) flow diagram [18] (Figure 2).

### 2.2. Search Strategy

The literature search was conducted using PubMed, Google Scholar, Scopus, and the Web of Science (WoS) databases. The WoS is a reputable source for documenting scientific studies across various disciplines [19,20]. The Boolean logical connector (OR) and truncation were applied in the search strategy, with the timelines being from 2015 to 2025. The search terms for PubMed and Google Scholar were “Food fraud in South Africa” OR “Food adulteration in South Africa “, OR “Food concealment in South Africa”, OR “Food Diversion in South Africa”, OR “Food theft in South Africa” OR “Food grey market in South Africa”, OR “Food dilution in South Africa, OR “Food mislabelling in South Africa, OR “Food counterfeit in South Africa”, OR “Food substitution in South Africa, OR “Food unapproved enhancement in South Africa”. The following search terms were used for Web of Science and Scopus: (Food fraud in South Africa) OR (Food adulteration in South Africa) OR (Food concealment in South Africa) OR (Food Substitution in South Africa) OR (Food Diversion in South Africa) OR (Food theft in South Africa) OR (Food grey market in South Africa) OR (Food dilution in South Africa) OR (Food mislabelling in South Africa) OR (Food counterfeit in South Africa) OR (Food substitution in South Africa) OR (unapproved food enhancement in South Africa). Search terms were adapted for each database.

### 2.3. Study Selection

All records were imported into an Excel extraction sheet and screened in two stages. First, titles and abstracts were reviewed for relevance, and duplicates were removed using EndNote. Two reviewers independently conducted the screening process and resolved disagreements through discussion. Full-text articles were then assessed against the eligibility criteria.

## 3. Results

A total of 853 (*n* = 853) articles or records were searched in three databases and other sources (*n* = 7); the two combined gave a total of 860 (*n* = 860). After accounting for duplication, the articles were further screened (*n* = 858). The remaining articles were screened for relevance and full-text availability (*n* = 89). The final step involved discarding articles unrelated to food fraud in South Africa; only 14 articles (*n* = 14) were included in the review, of which 2 articles were from the media, as shown in Figure 2.

### Food Fraud Studies in South Africa

Table 2 provides an overview of studies and reports on food fraud in South Africa. Studies that tested the authenticity of food products to prevent food fraud were included, as were fruits and vegetables found to contain excessive levels of chemicals during export to other countries, as well as cases reported by reputable news channels. Fourteen records were identified: three conducted experiments to test the authenticity of food products, and five involved sample collection. Study 1 examined undeclared meat species in processed meats; 40 processed meat samples were analysed, and 65% were contaminated with undeclared meat species. Study 2 examined 44 samples of ready-to-eat canned meat products. Notably, 12 (27.27%) of the 44 samples had undeclared meat species. Study 3 examined 77 samples of decapod crustaceans, and 4 (31%) were mislabelled or misrepresented. Study 4 examined 9 pure meat samples and 155 processed meat samples. Pure meat samples were contaminated with traces of other meat species (2%), and of the 155 processed meat samples, beef biltong was contaminated with 36% of pork. Study 5 examined 149 seafood samples; 18% of samples from restaurants were mislabelled, and 19% of samples from retail outlets were mislabelled or mispresented. Most studies indicate that food fraud is committed intentionally for economic gain by manufacturers or food-processing organisations. It included the substitution and mislabelling of food products, the replacement of sell-by dates, the sale of counterfeit products, the addition of chemicals to enhance flavour, the addition of pesticides, and illicit trade.

## 4. Discussion

Based on the list of food fraud activities identified in Table 2, further research is needed to formulate solutions relevant to the South African context. However, it is also worth noting that South Africa was not among the list of countries in Africa and worldwide in 2023 in the study by Polakova et al. [32], who gave an overview of food adulteration and listed Nigeria as one of the countries with high incidents of food adulteration mainly in alcoholic beverages, oils and fats, and grain-based products. Topping the list worldwide was Pakistan, with 59 food fraud cases. The discussion will focus on food adulteration categories prevalent in South Africa, including mislabelling, substitution, replacement of date markings, and counterfeit products.

### 4.1. Food Fraud Overview Across Different Categories

Fraud types such as substitution and counterfeiting exhibit both high economic motivation and high regulatory exploitation, while adulteration carries the highest public health risk. Mislabelling of food products is well documented, particularly in developed countries, with seafood products among the most commonly mislabelled [33,34]. A few studies conducted in South Africa [25,35,36,37,38] have found that fish products were misnamed. Some cases included lower-value fish species being named as higher-value species [39,40,41,42]. Therefore, consumers unknowingly pay more for a lower-value product, presented as a well-known brand or a high-value name or species [43]. It is essential to note that food product mislabelling can occur at any stage of the food supply chain. Although Khaksar et al. [34] found that restaurants had higher rates of mislabelling than retailers, they also cautioned that restaurants may themselves be victims, depending on the location of the mislabelling. Other instances could include cases in which butcheries use the same machinery to process different meat species without adequately cleaning them between species. Other meat products are mislabelled as beef products, and beef and mutton are contaminated with pork [21,35].

Research reports instances in which offenders, upon laboratory testing, were found to have tampered with food products. The horse meat scandal is one example of substituting one meat species with another. Patties made of horse meat were labelled as beef patties [5,7,44,45]. Similar incidents of false claims were reported in a study by Cawthorn et al. [35,36], which documented cases in which a product was marketed as a beef patty but contained horse and pork instead of beef. In some instances, pork meat was added to a meat product and labelled as beef [22,37]. Sunflower oil was labelled as pure olive oil, and honey was mixed with cane sugar or syrup but labelled as pure honey [5,29,46]. All these incidents justify a national testing strategy with robust analytical methods. These will form the backbone of routine surveillance. Analytical capability reduces exposure to toxic adulterants and undeclared allergens for vulnerable groups [6,47,48].

The practice of replacing date markings is a common form of malpractice, especially in products sold and labelled with sell-by dates. Current dates are removed and replaced with a label that extends the products’ sell-by date [14]. Such acts have dire consequences, as food contamination is inevitable when adherence to food control regulations is lacking, thereby compromising public health.

The South African food industry has been rocked by the mushrooming of factories manufacturing products containing unauthorised food colourants and/or additives and selling them to impoverished communities who buy food based on affordability [16,27]. These are cases in which food manufacturers or processors develop products that are, in most cases, of inferior quality and use packaging materials from well-known food brands. Examples include fire hydrant water being bottled in well-known water bottling companies’ bottles, indicating that the bottler’s packaging materials and labelling have been stolen. Water is sold to consumers under a familiar brand, without their awareness of what they are purchasing. There have been reported cases of non-alcoholic beverages being adulterated [49]. Spices are also among the products affected, according to a study by Velázquez et al. [50]. Beans and noodles were reported to be adulterated [51,52], and these products were packaged under the guise of well-known brands. Discovery of illicit liquor and food processing plants in the Gauteng region of South Africa [14,17]. These incidents could lead to the assumption that stringent enforcement of regulations is lacking or that environmental health practitioners responsible for food regulation are understaffed.

### 4.2. Regulatory Challenges

Food fraud can be viewed as a system shaped by opportunity, motivation, and control measures. Intentional fraud constitutes a criminal offence, whereas unintentional mislabelling is typically treated as regulatory non-compliance. This is denoted by van Ruth et al. [53], who provide a framework for designing surveillance indicators, e.g., for situations where opportunity is high, controls are weak, and motivation is elevated. The literature shows that food safety challenges in developing regions stem from both the misuse of chemical preservatives and widespread food fraud, enabled by weak and fragmented regulatory frameworks, limited technical capacity, insufficient enforcement capabilities, the lack of food fraud-specific laws, and limited consumer awareness [54]. Nnaji et al. [55] reported that harmful substances such as industrial dyes, pesticides, ripening agents, and formalin are frequently used due to economic pressures and low public awareness, exposing consumers to carcinogenic and neurotoxic risks. Similarly, Abid et al. [56] found that adulteration, dilution, mislabelling, and counterfeiting persist where oversight, surveillance, and laboratory systems are fragile, a situation worsened by socioeconomic constraints and pandemic-related disruptions. Although advanced detection tools such as spectroscopy, DNA barcoding, chromatography, and blockchain traceability could improve control, their uptake remains slow due to resource constraints in South Africa. In recent years, the South African government has been implementing a system to register all food-handling organisations in the informal sector and maintain a database. This was the approach that previously focused mainly on the formal food sector. Having a database of all food-handling organisations, both formal and informal, will facilitate the traceability of food products from the point of origin and enhance communication between the government and food-handling organisations. The implications of food fraud are threefold: consumer health and safety, enforcement of food regulations, and the industry’s reputation [4]. In South Africa, there are no food fraud databases comparable to the European Union’s Rapid Alert System for Food and Feed, the US FDA’s Economically Motivated Adulteration database, the USP Food Fraud database, the China National Food Safety Traceability Platform, the Food Standards Australia New Zealand database, and the Canada Food Inspection Agency database. The lack of a food fraud database in South Africa, as well as food safety laws to control food fraud and/or define it, contributes to the rise in food fraud incidents [3,13,17,52]. South African food safety regulatory laws under the National Department of Health consist mainly of the National Act, the Foodstuffs, Cosmetics, and Disinfectant Act, 54 of 1972 (FCD Act) [57], which governs the sale, manufacture, and importation of foodstuffs, cosmetics, and disinfectants. Section 2 of the Act indicates “any person shall be guilty of an offence if he or she sells, manufactures, or imports for sale any foodstuff (c) (ii) to which any substance has been added to increase the mass or volume of such foodstuff to deceive, or (iii) in which any substance or ingredient has been abstracted, removed or omitted with the result that its nutritive value or other properties, in comparison with those of such a foodstuff in a normal, pure and sound condition, are diminished or otherwise detrimentally affected.” This section indirectly addresses the substitution and dilution subcategories of food fraud. The FCD Act is supported by regulations promulgated under it, such as Regulation 638 of 2018 [58], a regulation governing general hygiene requirements for food premises, transport of food, and related matters; Regulation 504 of 2003 [59], a regulation relating to the fortification of certain foodstuffs; and Regulation 146 of 2010, a regulation relating to the labelling and advertising of foodstuffs, which indirectly addresses some of the subcategories of food fraud with provisions for permissible limits for food additives, preservatives, and date markings. As an example, Section 36(1) requires that all additives be listed in the ingredients list, which indirectly addresses the use of unapproved food additives [60]. The South African government urgently needs to revise food safety laws to keep pace with current practices and invest in the latest food authentication methods. It is well documented that traditional food safety hazard identification methods are unable to detect food fraud during production [24,34,37].

Few studies conducted in South Africa that have investigated food fraud have noted that inadequate enforcement of regulations and a lack of laboratory facilities are contributing factors to the increased incidence of food fraud [14,36,38,61]. Environmental health practitioners (EHPs) employed by the local municipalities are responsible for the enforcement of food safety laws under the Department of Health, which include monitoring compliance of all food handling organisations from informal food vendors, food retailers, restaurants, and food manufacturers to importers and exporters of food in and out of the country, to name a few, with the provision of the FCD Act and regulations promulgated under this Act [57]. According to the World Health Organisation (WHO), the staffing ratio for EHPs should be 1:10,000 (1 EHP per 10,000 people), as highlighted in the National Environmental Health Policy No. 951 of 2013 [62]. Table 3 illustrates South Africa’s shortfall in reaching this goal, showing the number of EHPs employed in each province compared with the number required for the given population. It is important to note that food fraud persists not only due to enforcement gaps but also to structural fragmentation in governance. There is a need to restructure environmental health service provision to meet the dynamic needs of communities and achieve the objective of protecting public health. South Africa’s environmental health system faces persistent challenges that undermine its effectiveness. Mbazima et al. [63] highlighted that weaknesses such as a shortage of qualified practitioners, inadequate funding, inconsistent training standards, and limited public awareness have left the profession struggling to deliver essential services. They further asserted that research capacity and data-driven approaches remain underdeveloped, while the profession itself is often misunderstood and undervalued compared to curative health services. Although supportive legislation, political stability, and knowledge-sharing platforms have strengths, these are outweighed by systemic weaknesses and threats. The lack of enforcement capacity facilitates the continued occurrence of food fraud [64,65,66].

#### Penalties for Offenders

Examples of legal consequences for intentional food fraud highlight significant disparities between jurisdictions. In the United States, penalties have included substantial fines and imprisonment: for instance, in 2019 and 2020, the father-and-son owners of Casey’s Seafood were sentenced to prison for repackaging foreign crab meat as U.S. blue crab, while the owner of Capt. Neill’s Seafood was sentenced to prison and ordered to pay a $250,000 fine, and the company was placed on probation and fined $500,000 [68]. Similarly, in 2016, the president of Castle Cheese was sentenced to three years’ probation, fined $5000, and ordered to complete 200 h of community service for selling grated Parmesan adulterated with cellulose and cheddar labelled as 100% Parmesan cheese [69]. In Canada, a farm was fined $1.5 million for mislabelling Mexican-grown produce as Canadian, and two executives were prosecuted alongside the company [69]. In contrast, in South Africa, penalties under the FCD Act are minimal and not specific to food fraud: Section 18(1) prescribes fines of up to R400 ($24.60 USD) or six months’ imprisonment for a first conviction, R800 ($48.72 USD) or twelve months for a second, and R2000 ($121.79 USD) or 24 months for a third offence. These penalties are unlikely to deter offenders, particularly when compared to international standards. Furthermore, South Africa lacks legislation specifically addressing food fraud, which would provide clear definitions and categories of fraudulent activities [4,5,64]. Stronger, targeted laws and harsher penalties are essential to ensure compliance and convey the seriousness of food fraud within the industry [70]. This is strongly supported by Momtaz et al. [51] and Van Ruth et al. [53], who advocate specific policy instruments, including import controls, additive controls, labelling enforcement, and deterrent penalties.

### 4.3. Communities at Risk

Food fraud, whether intentional or unintentional, has profound implications for consumers’ health. There is a likelihood of allergic reactions, which could lead to the death of atopic or hypersensitive individuals. It undermines human rights, people’s religious beliefs, and their freedom of choice [24,33,36,71]. Individuals from lower socioeconomic classes, children, and those without education are at risk, as they often purchase food based on the affordability of products. Communities in rural areas are more at risk of food fraud due to limited access to media coverage and information about food fraud [16,17].

**Figure 3:**
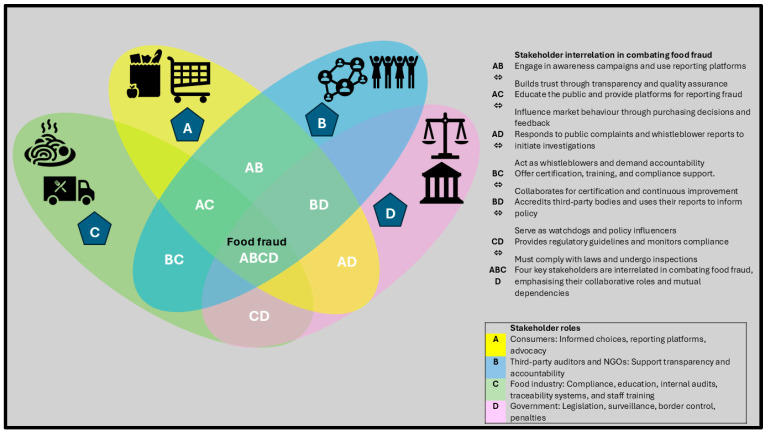
Model illustration of a dynamic ecosystem for stakeholder interaction in combating food fraud.

Food safety is everyone’s responsibility [72,73]. To combat food fraud, government departments, the food industry, and consumers must collaborate. Each unit plays a role [48]. The government must ensure regular updates on food laws, stringent enforcement, and education and awareness of these laws to both consumers and the food industry [54,73,74]. The food industry has a responsibility to comply with established regulations and to educate consumers about its products, including product authenticity. The consumer’s role is to follow the manufacturer’s instructions for the intended use of food products and to report any wrongdoing or suspicious activity to the food industry and law enforcement. This is illustrated in Figure 3.

### 4.4. Food Industry Role

The food industry is expected to uphold food safety standards. In addition to food safety management systems required for the food supply chain, it must also consider ways to mitigate food fraud, such as blockchain technology, artificial intelligence, and food fraud detection methods, as well as intelligent packaging, food authentication techniques, and anti-counterfeiting and fraud mitigation solutions [12,71,74]. Adoption of these systems will make it difficult for fraudsters to replicate their packaging and tamper with their products. The challenge with these systems is that they are costly and need highly specialised personnel to operate. Partnerships with academia may help develop cost-effective analytical and authentication methods for food. However, day-to-day operational strategies must be implemented to mitigate food fraud. Food handling organisations can use the food fraud risk factors recommended by Hoffman et al. [6]. These include vetting and certifying suppliers. They also involve understanding the nature of the product, since powders and liquids are easier to tamper with than solid products. Another factor is the investment in employee training in food safety and quality principles [75,76]. Employees who feel valued can become brand ambassadors. Raw material vulnerability is also important, as high-value products such as honey and olive oil are susceptible to adulteration. Production and processing steps must be examined because vulnerable points can increase the risk of food fraud. Traceability of products is critical; keeping the supply chain short facilitates management, which can be achieved by adopting a food fraud vulnerability assessment tool developed by the SSAFE organisation [77]. Finally, market characteristics, influenced by economic and political pressures, may raise the risk of food adulteration. To mitigate the identified risks, Spink et al. [15] recommended a similar approach to HACCP, in which food-handling organisations must assemble a food fraud team trained in food fraud principles and vulnerability assessment. Incorporate food fraud and mitigation plans in the organisation’s food safety policy. The assembled food fraud team must identify all types of food fraud risks and determine the appropriate mitigation controls.

### 4.5. Recommendations

To effectively combat food fraud and enhance food safety, a comprehensive database of all food-handling organisations should be created and regularly updated. In addition, a publicly accessible food fraud database must be established [78], supported by awareness campaigns to educate stakeholders on its purpose and benefits. The food industry should invest in initiatives that promote product authentication, such as ScanTrust, which uses QR codes to verify product authenticity and origin, and invest in non-invasive authentication methods [27,79]. Rapid detection techniques for commonly adulterated food products in South Africa should be developed alongside targeted communication strategies to reach vulnerable groups, including rural communities, schoolchildren, and individuals from low socioeconomic backgrounds who often prioritise affordability over quality. While advanced technologies show promise, scalable, low-cost detection strategies and institutional strengthening remain immediate priorities in South Africa. In addition to rapid detection techniques, where evidence is fragmented and a coordinated database is lacking, as in South Africa, predictive tools can still guide targeted monitoring. At the same time, systems mature, as Rezazade et al. [78] have shown in similar scenarios.

Rigorous inspection of products before market placement is essential, complemented by the employment of environmental health practitioners in line with the 1:10,000 population ratio stipulated under environmental health norms and standards. Furthermore, specific regulations addressing food fraud-related crimes must be introduced, with harsher penalties to deter offenders. Strict enforcement of these regulations should include routine and targeted sampling programmes, as well as interdepartmental collaboration to strengthen detection and prevention efforts, particularly for imported goods. Given that food fraud evolves with new regulations and detection methods, monitoring processes must be continuously adapted [80]. Raising awareness among food industry members about common fraudulent commodities and prevention measures is crucial, alongside mandatory testing and certification to confirm authenticity. While current detection methods may be costly, developing more efficient and accessible techniques is imperative. Finally, fostering partnerships among government (harmonisation of safety laws and implementation thereof), industry, and academia will be key to addressing this issue effectively, as inadequate penalties have historically failed to deter [48].

## 5. Limitations

This review has several limitations that must be acknowledged when interpreting the findings. Although the search strategy included reputable scientific databases (Web of Science Core Collection, Scopus, and PubMed), Google Scholar, and reliable media sources, publication bias remains a concern, as many food fraud incidents, especially those uncovered by regulatory authorities, consumer complaints, or routine inspections, are not published in peer-reviewed literature. Fraudulent activities are often concealed by perpetrators or resolved administratively without formal documentation, resulting in an incomplete representation of the true extent of the problem in South Africa. A further limitation concerns the underreporting of food fraud in informal markets, which constitute a significant component of South Africa’s food economy. These markets are frequently unregulated, lack systematic inspection, and operate outside established surveillance frameworks. Consequently, many counterfeit, adulterated, or mislabelled products circulating in these settings remain undocumented, limiting the data available for academic synthesis. The review also reflects the limited local research output on food fraud and the absence of a coordinated national database or surveillance system to track incidents. Existing studies are scattered across different food categories and geographic regions, with minimal longitudinal assessments. This fragmented evidence base restricts the ability to analyse trends over time or comprehensively evaluate the scale of fraud across the national supply chain.

Future research will need to integrate statistically robust sampling frameworks, transparent reporting of analytical validation, and access to routine surveillance data to reduce these biases and provide a more accurate picture of the national food fraud burden.

## 6. Conclusions

This review demonstrates that food fraud in South Africa is a pervasive, multifaceted, and increasingly sophisticated challenge with profound implications for public health, regulatory governance, and consumer protection. Evidence from the past decade highlights intentional adulteration, mislabelling, substitution, counterfeit production, and illicit trade across multiple commodity groups. Driven by economic incentives, these practices undermine consumer trust, compromise food safety, and expose vulnerable populations to harmful chemicals, undeclared allergens, pathogenic contaminants, and misrepresented ingredients. While some studies have explored authentication technologies and detection methods, the overall scientific landscape remains limited, revealing a significant national research gap relative to global advances.

The review highlights systemic regulatory shortcomings, including inadequate enforcement capacity, insufficient laboratory infrastructure, the absence of a national food fraud surveillance database, and outdated legislative penalties that fail to deter offenders. Strengthening the national response requires a coordinated, multisectoral approach that foregrounds prevention, rapid detection, and accountability. This includes establishing a national reporting and monitoring system, enhancing laboratory and inspection capacity, adopting advanced authentication tools, and implementing stricter regulations. Collaborative action among government, industry, academia, and civil society, supported by investments in research, improved traceability, targeted surveillance, and public awareness, will be essential to protect consumer health, uphold the integrity of the food supply, and contribute to global efforts to combat economically motivated adulteration.

## Figures and Tables

**Table 2:** Food fraud cases in South Africa.

Study ID	Food Category	Type of Fraud	Key Findings/Examples	Intent	Source
1	Processed meat (beef sausages, beef patties, chicken sausages, and chicken patties)	Species substitution, mislabelling	Undeclared pork, mutton, chicken, and other products were detected in products labelled as beef.	* Intentional and ** unintentional	[21]
2	Canned and ready-to-eat meat	Substitution	Products marked with “no pork” claim tested positive for pork. Some products tested positive for chicken, beef, or sheep, which are not declared on the labels.	* Intentional	[22]
3	Seafood (finfish, shrimps, prawns, crabs)	Mislabelling, misrepresentation	Labelled species are inconsistent with DNA authentication. Misleading labelling of prawns. In contravention of South African food labelling regulations.	* Intentional and ** unintentional	[23]
4	Honey Packaged foods (beans, noodles, cereals) Olive oil Non-alcoholic beverages Alcoholic beverages, chicken	Adulteration, counterfeit, substitution. Addition of chemicals/unauthorised additives (E-104) Unauthorised additives (methanol). Mislabelling—placing new expiration dates on chicken products when the original expiration dates are reached.	Addition of cane sugar/corn syrup Replication of brand packaging: inferior ingredients used. Sunflower or blended oils are sold as pure olive oil. Fire hydrant water bottled using a well-known brand’s packaging.	* Intentional	[14]
5	Processed meat products: mincemeat, sausages, biltong, burger patties	Replacing/mislabelling/substitution	Replacing/mislabelling/substitution, e.g., mislabelling pork sausage as beef sausage. Addition of plant protein to patties Pork sausage mixed with beef, pork, and Mutton	* Intentional -Economic gain/reduce production costs	[24]
6	Fish species: Finfish (kingklip, hake, kabeljou, tuna	Substitution and mislabelling/misnaming.	Samples were genetically identified as belonging to species different from those indicated at the point of sale.	* Intentional and ** unintentional	[25]
7	Fruits and vegetables	Chemical adulteration	Excessive pesticide residues (ethephon, oxamyl, methamidophos, cetrimonium chloride) flagged during export screening.	* Intentional/Serious risk	[26]
8	Abalone (seafood)	Grey-market trade	Illegal harvesting, smuggling, and misrepresentation of origin	* Intentional	[17]
9	Spices, herbs, and yeast	Counterfeit	Fake branded spices detected in informal markets	* Intentional	[27]
Laboratory-based authenticity and method-development studies					
10	Beef Patty	Not applicable	Tested a method for the authentication of raw beef patties. Analysing near-infrared hyperspectral imaging system as an alternative authentication technique (NIR-HIS)	Authentication of food products	[28]
11	Beef products	Not applicable	Tested a method for the authentication of beef products. Testing the authenticity of mincemeat using near-infrared and mid-infrared spectroscopy.	Authentication of meat products within the meat industry	[9]
12	Honeybush and rooibos	Not applicable	Tested a method for the authentication of rooibos and honeybush. Testing authenticity using energy-dispersive X-ray fluorescence spectrometry (EDXRF) and different one-class classification techniques.	Authentication of honeybush and rooibos tea	[29]
Incidents Reported through reputable media platforms in South Africa					
13	Eggs and fish	Mislabelling and adulteration	Misrepresentation of caged hens as free range, and adding colourants to hake and labelled as salmon.	* Intentional	[30]
14	Honey	Dilution and mislabelling	Adulteration with fructose Syrup. Importing cheap bulk honey and selling it as a product of South Africa.	* Intentional	[31]

* Intentional fraud = deliberate economic deception. ** Unintentional fraud = cross-contamination, labelling errors.

**Table 3:** Environmental health staffing-to-population ratio [3,67].

Province	EHPs in Public Service in 2023	Required EHPs Per WHO Ratio of (1:10,000)	Population by Statistics SA 2023
Eastern Cape	203	723	7,230,204
Free State	71	296	2,964,412
Gauteng	437	1510	15,099,422
KwaZulu Natal	287	1242	12,423,907
Limpopo	129	657	6,572,721
Mpumalanga	81	514	5,143,324
Northwest	76	380	3,804,548
Northern Cape	55	136	1,355,945
Western Cape	373	743	7,433,020

## Data Availability

The original contributions presented in the study are included in the article; further inquiries can be directed to the corresponding authors.

## References

[1] Brooks C., Parr L., Smith J.M., Buchanan D., Snioch D., Hebishy E. (2021). A review of food fraud and food authenticity across the food supply chain, with an examination of the impact of the COVID-19 pandemic and Brexit on food industry. Food Control.

[2] Spink J., Moyer D.C. (2011). Defining the public health threat of food fraud. J. Food Sci..

[3] Chukwugozie D.C., Njoagwuani E.I., David K., Okonji B.A., Milovanova N., Akinsemolu A.A., Ghosh S. (2024). Combatting food fraud in sub-Saharan Africa: Strategies for strengthened safety and security. Trends Food Sci. Technol..

[4] Robson K., Dean M., Haughey S., Elliott C. (2021). A comprehensive review of food fraud terminologies and food fraud mitigation guides. Food Control.

[5] Spink J., Chen W., Zhang G., Speier-Pero C. (2019). Introducing the food fraud prevention cycle (FFPC): A dynamic information management and strategic roadmap. Food Control.

[6] Hoffman L.C., Schreuder J., Cozzolino D. (2025). Food authenticity and the interactions with human health and climate change. Crit. Rev. Food Sci. Nutr..

[7] Viscoano P., Schirone M. (2021). Food frauds: Global incidents and misleading situations. Trends Food Sci. Technol..

[8] Ehmke M.D., Bonanno A., Boys K., Smith T.G. (2019). Food fraud: Economic insights into the dark side of incentives. Aust. J. Agric. Resour. Econ..

[9] Aslam R., Sharma S.R., Kaur J., Panayampadan A.S., Dar O.I. (2023). A systematic account of food adulteration and recent trends in the non-destructive analysis of food fraud detection. J. Food Meas. Charact..

[10] Edwards K., Manley M., Hoffman L.C., Williams P.J. (2021). Non-destructive spectroscopic and imaging techniques for the detection of processed meat fraud. Foods.

[11] Choudhary A., Gupta N., Hameed F., Choton S. (2020). An overview of food adulteration: Concept, sources, impact, challenges and detection. Int. J. Chem. Stud..

[12] Meerza S.I.A., Giannakas K., Yiannaka A. (2019). Markets and welfare effects of food fraud. Aust. J. Agric. Resour. Econ..

[13] Tang A., Tchao E.T., Agbemenu A.S., Keelson E., Klogo G.S., Kponyo J.J. (2024). Assessing blockchain IoT technologies for agricultural food supply chain in Africa: A feasibility analysis. Heliyon.

[14] Boller M.L., Zurwehme A., Krupitzer C. (2024). Qualitative assessment on the chances and limitations of food fraud prevention through distributed ledger technologies in the organic food supply chain. Food Control.

[15] Mphaga K.V., Moyo D., Rathebe P.C. (2024). Unlocking food safety: A comprehensive review of South Africa’s food control and safety landscape from an environmental health perspective. BMC Public Health.

[16] Mbonane T., Rathebe P. (2019). Fake food’ epidemic in South Africa: A growing food safety/public health issue?. S. Afr. J. Public Health.

[17] Soon-Sinclair J.M., Imathiu S., Obadina A.O., Dongho Dongmo F.F., Kamgain A.D.T., Moholisa E., Saba C.K.S., Walekhwa A.W., Hunga H., Kussaga J. (2023). How Worried Are You about Food Fraud? A Preliminary Multi-Country Study among Consumers in Selected Sub-Saharan African Countries. Foods.

[18] Moher D., Liberati A., Tetzlaff J., Altman D.G., Group P. (2009). Preferred reporting items for systematic review and meta-analyses: The PRISMA statement. Ann. Intern. Med..

[19] Li K., Rollins J., Yan E. (2018). Web of Science use in published research and review papers 1997–2017: A selective, dynamic, cross-domain, content-based analysis. Scientometrics.

[20] Secinaro S., Calandra D. (2020). Halal food: Structured literature review and research agenda. Brit. Food J..

[21] Tembe D., Mukaratirwa S., Zishiri O.T. (2018). Undeclared Meat Species in Processed Meat Products from Retail Franchises in the Durban Metropole, KwaZulu-Natal Province, South Africa, Using Species-specific DNA Primers. Food Prot. Trends.

[22] Sreenivasan Tantuan S., Viljoen C.D. (2021). Determining the presence of undeclared animal species using Real-time PCR in canned and ready-to-eat meat products in South Africa. J. Food Sci. Technol..

[23] Cawthorn D.M., Hoffman L.C. (2017). Deceit with decapods? Evaluating labelling accuracy of crustacean products in South Africa. Food Control.

[24] Chaora N.S., Khanyile K.S., Magwedere K., Pierneef R., Tabit F.T., Muchadeyi F.C. (2022). A 16S next-generation sequencing-based molecular and bioinformatics pipeline to identify processed meat products contamination and mislabelling. Animals.

[25] Cawthorn D.M., Duncan J., Kastern C., Francis J., Hoffman L.C. (2015). Fish species substitution and misnaming in South Africa: An economic, safety and sustainability conundrum revisited. Food Chem..

[26] RASFF (2024). RASFF Window, European Commission. https://webgate.ec.europa.eu/rasff-window/screen/search.

[27] Mofokeng N.E.M. (2018). Safeguarding township tourism in South Africa from counterfeit consumable products through consumer oriented technological solutions. Afr. J. Hosp. Tour. Leis.

[28] Edwards K., Hoffman L.C., Manley M., Williams P.J. (2023). Raw beef patty analysis using near-infrared hyperspectral imaging: Identification of four patty categories. Sensors.

[29] Malyjurek Z., Zawisza B., De Beer D., Joubert E., Walczak B. (2021). Authentication of honeybush teas based on their elemental composition. Food Control.

[30] The Media Online (2016). Mislabeling Concerns in SA. https://themediaonline.co.za/2016/01/mislabeling-concerns-in-sa/.

[31] Knowler W. (2021). Falsely Labelled, Mixed with Syrup or ‘Laundered’: Honey Fraud Is Rife in SA. Sunday Times: South Africa. https://www.timeslive.co.za/news/consumer-live/2021-05-21-falsely-labelled-mixed-with-syrup-or-laundered-honey-fraud-is-rife-in-sa/.

[32] Poláková K., Bobková A., Demianová A., Bobko M., Jurčaga L., Mesárošová A., Čapla J., Timoracká I., Lidiková J., Čeryová N. (2024). Adulteration in food industry in 2023-overview. J. Microbiol. Biotechnol. Food Sci..

[33] Giusti A., Malloggi C., Tinacci L., Nucera D., Armani A. (2023). Mislabeling in seafood products sold on the Italian market: A systematic review and meta-analysis. Food Control.

[34] Khaksar R., Carlson T., Schaffner D.W., Ghorashi M., Best D., Jandhyala S., Traverso J., Amini S. (2015). Unmasking seafood mislabeling in US markets: DNA barcoding as a unique technology for food authentication and quality control. Food Control.

[35] Cawthorn D.M., Steinman H.A., Hoffman L.C. (2013). A high incidence of species substitution and mislabelling detected in meat products sold in South Africa. Food Control.

[36] Cawthorn D.M., Steinman H.A., Witthuhn R.C. (2012). DNA barcoding reveals a high incidence of fish species misrepresentation and substitution on the South African market. Food Res. Int..

[37] Bannor R.K., Arthur K.K., Oppong D., Oppong-Kyeremeh H. (2023). A comprehensive systematic review and bibliometric analysis of food fraud from a global perspective. J. Agric. Food Res..

[38] Nelwamondo V. (2023). A Stakeholder Assessment of the Food Fraud Vulnerability of the South African Meat Sector: A Case Study of the Tshwane Metropolitan Area. https://repository.up.ac.za/items/b92f3841-273b-4c91-ac55-8ecbc8922568.

[39] Montgomery H., Haughey S.A., Elliott C.T. (2020). Recent food safety and fraud issues within the dairy supply chain (2015–2019). Glob. Food Secur..

[40] Lawrence S.J., Elliott C., Huisman W., Dean M., van Ruth S. (2022). The 11 sins of seafood: Assessing a decade of food fraud reports in the global supply chain. Comp. Rev. Food Sci. Food Saf..

[41] Kyrova V., Surmanova P., Ostry V., Rehurkova I., Ruprich J., Jechova M. (2017). Sea fish fraud? A confirmation of Gadoid species food labelling. Brit. Food J..

[42] Jurica K., Brčić Karačonji I., Lasić D., Bursać Kovačević D., Putnik P. (2021). Unauthorised Food Manipulation as a Criminal Offence: Food Authenticity, Legal Frameworks, Analytical Tools and Cases. Foods.

[43] Ham J.-H., Lee Y.-J., Lee S.-S., Kim H.-Y. (2025). Food Fraud in Plant-Based Proteins: Analytical Strategies and Regulatory Perspectives. Foods.

[44] Alewijn M., Goemans P., Gussow K.E., Turner K.J., Pustjens A.M. (2025). An Analysis of the Severity of Food Safety Hazards in EU Food Fraud Cases. Foods.

[45] Watters S. (2015). The Spectacular Origins of the EU Horse Meat Scandal. Graduate J. Food Stud..

[46] Bimbo F., Bonanno A., Viscecchia R. (2019). An empirical framework to study food labelling fraud: An application to the Italian extra-virgin olive oil market. Aust. J. Agric. Res. Econ..

[47] Callao M.P., González A.G. (2018). Analytical Methods to Combat Food Fraud: A Review. Food Control.

[48] Manning L., MacLeod A., James C., Thompson M., Oyeyinka S., Cowen N., Skoczylis J., Onarinde B.A. (2024). Food fraud prevention strategies: Building an effective verification ecosystem. Comp. Rev. Food Sci. Food Saf..

[49] Syposs Z., Knight G., Hellberg R.S., Everstine K., Sklare S.A. (2021). Fraud in Nonalcoholic Ready-to-Drink Products. Food Fraud: A Global Threat with Public Health and Economic Consequences.

[50] Velázquez R., Rodríguez A., Hernández A., Casquete R., Benito M.J., Martín A. (2023). Spice and Herb Frauds: Types, Incidence, and Detection: The State of the Art. Foods.

[51] Momtaz M., Bubli S.Y., Khan M.S. (2023). Mechanisms and Health Aspects of Food Adulteration: A Comprehensive Review. Foods.

[52] Njiru J.M., Njeru E., Kang’iri J., Lunani I., Rotich H., Muriira G., Tombito C., Nyaboga E.N. (2025). Food fraud in selected sub-Saharan Africa countries: A wake-up call to national regulatory bodies to support enforcement and food safety. Front. Food Sci. Technol..

[53] van Ruth S.M., Huisman W., Luning P.A. (2018). Food Fraud Vulnerability and Its Key Factors. Food Control.

[54] Abid H.M.R., Asam S., Awan N., Khad N. (2025). Current Readiness on Food Fraud Risk Mitigation in Developing Countries: A Review. Agric. Food Secur..

[55] Nnaji N.D., Onyeaka H., Ughamba K.T., Ononugbo C.M., Olovo C.V., Mazi I.M. (2025). Chemical Toxicants Used for Food Preservation in Africa: Is It a Case of Ignorance or Food Fraud?. Health Sci. Rep..

[56] Crisan E. (2025). Food Fraud—A Priority for EU Food Law. Eur. J. Comp. Law Gov..

[57] South Africa (1972). Foodstuffs, Cosmetics and Disinfectants Act, Act No.54 of 1972. Government Printers, Pretoria. https://www.gov.za/documents/foodstuffs-cosmetics-and-disinfectants-act-2-jun-1972-0000.

[58] (2018). South Africa. Regulations Governing General Hygiene Requirements for Food Premises, the Transport of Food and Related Matters, Government Gazette No. 41730, Regulations No. 638 of 22 June 2018. Government Printers, Pretoria. https://www.gov.za/documents/notices/foodstuffs-cosmetics-and-disinfectants-act-regulations-general-hygiene-0.

[59] (2003). South Africa. Regulations Relating to the Fortification of Certain Foodstuffs, Government Gazette No. 24715, Regulations No.504 of 7 April 2003. Government Printers, Pretoria. https://www.gov.za/sites/default/files/gcis_document/201409/315841206.pdf.

[60] (2010). South Africa. Regulations Relating to the Labelling and Advertisement of Foodstuffs, Government Gazette No.32975, Regulations No.146 of 1 March 2010. Government Printers, Pretoria. https://www.gov.za/sites/default/files/gcis_document/201409/32975146.pdf.

[61] Gizaw Z. (2019). Public health risks related to food safety issues in the food market: A systematic literature review. Environ. Health Prev. Med..

[62] South Africa (2013). South Africa. National Environmental Health Policy, No.951 of 2013. Government Printers, Pretoria. https://www.gov.za/documents/notices/national-environment-health-policy-04-dec-2013.

[63] Mbazima S.J., Mbonane T.P., Masekameni M.D. (2022). A SWOT analysis of contemporary gaps and a possible diagnostic tool for environmental health in an upper-middle-income country: A case study of South Africa. Int. J. Environ. Health Res..

[64] Cadieux B., Goodridge L.D., Spink J. (2019). Gap analysis of the Canadian food fraud regulatory oversight and recommendations for improvement. Food Control.

[65] Parliamentary Monitoring Group Question NW4197 to the Minister of Health, December 2023. https://pmg.org.za/committee-question/24463/.

[66] Pardo M.Á., Jiménez E. (2020). DNA barcoding revealing seafood mislabeling in food services from Spain. J. Food Compo. Anal..

[67] Statistics South Africa Department: Statistics South Africa, Republic of South Africa. Census 2022. https://census.statssa.gov.za/#/.

[68] U.S. Food and Drug Administration (.gov) Economically Motivated Adulteration (Food Fraud). https://www.fda.gov/food/compliance-enforcement-food/economically-motivated-adulteration-food-fraud.

[69] Food Fraud Advisors Food Fraud Risk Information. https://trello.com/b/aoFO1UEf/food-fraud-risk-information.

[70] Charlebois S., Schwab A., Henn R., Huck C.W. (2016). Food fraud: An exploratory study for measuring consumer perception towards mislabeled food products and influence on self-authentication intentions. Trends Food Sci. Technol..

[71] Donlan C.J., Luque G.M. (2019). Exploring the causes of seafood fraud: A meta-analysis on mislabeling and price. Mar. Pol..

[72] (2022). WHO Global Strategy for Food Safety 2022–2030: Towards Stronger Food Safety Systems and Global Cooperation. World Health Organisation (WHO). https://iris.who.int/server/api/core/bitstreams/c54caa7b-f6a7-4121-b9da-ee567198f4f8/content.

[73] Bouzembrak Y., Marvin H.J.P. (2016). Prediction of food fraud type using data from Rapid Alert System for Food and Feed (RASFF) and Bayesian network modelling. Food Control.

[74] Onyeaka H., Ukwuru M., Anumudu C., Anyogu A. (2022). Food fraud in insecure times: Challenges and opportunities for reducing food fraud in Africa. Trends Food Sci. Technol..

[75] Joenperä J., Lundén J. (2024). Food fraud detection and reporting by food control officers in Finland. Int. J. Environ. Health Res..

[76] Shi S., Zhang K., Tian N., Jin Z., Liu K., Huang L., Tian X., Cao C., Zhang Y., Jiang Y. (2025). Spectroscopic techniques combined with chemometrics for rapid detection of food adulteration: Applications, perspectives, and challenges. Food Res. Int..

[77] SSAFE Food Fraud Vulnerability Assessment Tool. https://www.ssafe-food.org/resources/food-fraud-vulnerability-assessment-tool.

[78] Rezazade F., Summers S., Ong K. (2022). Bayesian Network Modelling of Food Fraud Vulnerability Factors Using a Food Fraud Database. Food Control.

[79] Horns A.L., Barmbold S., Weidner M., Bachmann R. (2025). Spatially offset Raman spectroscopy (SORS) for sustainable olive oil authentication-tackling the challenges in on-site food control. Food Res. Int..

[80] Maritano V., Barge P., Biglia A., Comba L., Ricauda Aimonino D., Tortia C., Gay P. (2024). Anticounterfeiting and Fraud Mitigation Solutions for High-value Food Products. J. Food Prot..

